# Supplementary material for: A self-supervised domain-general learning framework for human ventral stream representation
Source: Nat Commun. 2022 Jan 25;13:491. doi: 10.1038/s41467-022-28091-4 (PMC8789817; doi:10.1038/s41467-022-28091-4)
Supplement: Supplementary file 1 — Supplementary Information [file 41467_2022_28091_MOESM1_ESM.pdf]

Supplementary Information

A self-supervised domain-general learning framework for human ventral stream representation

Talia Konkle and George A. Alvarez

1 Supplementary Methods: Modeling

1.1 Instance Prototype Contrastive Learning

In contrastive-learning frameworks, the goal is to learn an embedding function that maps images into a low-dimensional latent space, where visually similar images are close to each other, and visually dissimilar images are far apart. Learning proceeds by organizing the training data into similar pairs (positive samples) and dissimilar pairs (negative samples), where different frameworks make different choices of how positive and negative samples are encoded and retained throughout the learning process.

In our instance-prototype contrastive learning framework, we randomly augment the same image (x) multiple times ($n = 5$ in the models reported here), then pass each augmented image ($x_i \dots x_j$) through an embedding function $f_\theta(x)$ to obtain a low-dimensional representation of each image ($z_i \dots z_j$). We then compute an instance prototype $\bar{z}$ by averaging the embedding for all 5 samples:

$$\bar{z} = \frac{1}{n}\sum_{i=1}^{n} f_\theta(x_i) \tag{1}$$

where n is the number of samples, $f_\theta(x)$ is the embedding function (e.g., Alexnet-gn), and x_i is the ith augmented sample of an image.

For each augmented instance, the prototype serves as its positive pair, and all stored representations serve as negative pairs (implemented with a lightweight, non-indexed memory queue storing the $K = 4096$ most recent samples). The normalized temperature-scaled cross entropy loss for a positive pair $(z_i, \bar{z})$ would be defined as:

$$\ell_{z_i,\bar{z}} = -\log \frac{\exp(\text{sim}(z_i, \bar{z})/\tau)}{\exp(\text{sim}(z_i, \bar{z})/\tau) + \sum_{k=1}^{K} \exp(\text{sim}(z_i, z_k)/\tau)} \tag{2}$$

where the similarity function sim is the dot product between $L2$-normalized embeddings, τ is a temperature parameter that controls the dynamic range of the similarity function, and K is the total number of samples stored in the memory queue.

In practice, we used Noise Contrastive Estimation (NCE, Gutmann and Hyvärinen, 2010) to approximate sampling from a larger memory store (see Wu et al., 2018) though recent work suggests the loss function in equation 2 may suffice (Chen et al., 2020a). Specifically, we used Wu et al. (2018)'s implementation of Noise Contrastive Estimation to approximate sampling, with slight modifications to accommodate our prototype and queue:

$$\ell_{z_i,\bar{z}} = -(\log(Pos) + \log(Neg)) \tag{3}$$

$$Pos = \frac{\exp(\text{sim}(z_i, \bar{z})/\tau)/Z}{\exp(\text{sim}(z_i, \bar{z})/\tau)/Z + \frac{K}{N} + \epsilon} \tag{4}$$

$$Neg = \frac{\frac{K}{N}}{\sum_{k=1}^{K} [\exp(\text{sim}(z_i, q_k)/\tau)/Z + \frac{K}{N} + \epsilon]} \tag{5}$$

where z_i is the embedding for the ith sample, $\bar{z}$ is its corresponding prototype, sim is the similarity function (dot-product between embeddings), τ is the temperature parameter, Z is a normalization

constant (estimated based on the first mini-batch of 128×5 augmented samples), q_k is the embedding for the kth item stored in the queue, and $\epsilon = 1e - 7$ is a constant added for numerical stability.

The final loss is computed across all positive pairs in a minibatch (128 images, 5 samples per image, yielding 640 positive pairs). The queue is updated after every minibatch with the current samples added to the queue, displacing the oldest samples.

A PyTorch implementation and pretrained models for IPCL can be found at `https://github.com/harvard-visionlab/open_ipcl`.

1.2 Model Architecture Details

We created a modified AlexNet-gn architecture following the original AlexNet implementation from Krizhevsky et al. (2012) with three noteworthy differences: (1) We used *group normalization* (Wu and He, 2018) with 32 groups per layer, instead of local response normalization with 5 channels per group (Krizhevsky et al., 2012). (2) For the self-supervised models, the final 1000-way output layer was replaced with a fully-connected low-dimensional embedding space (128 dimensions), followed by an L2-normalization layer necessary for the contrastive learning task. (3) The original AlexNet's conv2, conv4, and conv5 layers were split across 2 GPUs for practical reasons — at the time GPUs had less RAM and could not fit the full model. While this split architecture can be emulated in PyTorch using grouped convolutional layers, we did not split the model architecture in this way because modern GPUs can fit the full model. Note the architecture details of this Alexnet are different from the official PyTorch version of AlexNet (`https://pytorch.org/vision/stable/models.html`), which implements Krizhevsky (2014). **Supplementary Figure 1** shows the exact model architecture specification for Alexnet-gn.

1.3 Image Augmentation Details

Our primary models were trained with the following augmentations ("Aug Set1"): (1) RandomResized-Crop, which grabs a random crop from the original image with the scale restricted to (0.2,1.) times the area of the original image and an aspect ratio in the range (3/4,4/3) times the aspect ratio of the original image, and then this cropped image was resized to 224x224 pixels. (2) HorizontalFlip with probability=.5, (3) conversion to GrayScale with probability=.2, (4) RandomColorJitter which adjusted the brightness, contrast, and saturation between (.6,1.4) times the original, and hue between +/- 144 degrees of the original image. As is standard, images were also normalized by z-scoring each pixel (i.e., subtracting the mean and dividing by the standard deviation for each channel). Unless otherwise noted, we used the standard Imagenet normalization parameters (mean=[0.485, 0.456, 0.406], std= [0.229, 0.224, 0.225]).

For models varying the visual diet we used a slightly different augmentation setting ("Aug Set2"). Specifically, we reduced the RandomResizedCrop scale range to (0.5,1.0) because the face images were already relatively zoomed in. We also standardized the normalization parameters (mean=[0.5, 0.5, 0.5], std= [0.2, 0.2, 0.2]) to apply the same normalization to each dataset (ImageNet, OpenImagesV6, Places2, and VGGFace2).

Our models were trained with custom data-augmentation functions that operate on the GPU to accelerate augmentation.

1.4 Training Details

Models were trained for 100 epochs with a batch size of 128x5 (128 images each augmented 5 times) using stochastic gradient descent, with momentum=.9 and weight decay=5e-4. Gradients were

accumulated across 20 batches before each optimizer step. The learning rate was varied using the one-cycle policy (Smith, 2017), beginning at 0.00003, increasing with a cosine annealing function to a maximum of .03 after 40 epochs, then decreasing with a cosine annealing function toward zero (3e-09) by 100 epochs. As part of a hyperparameter search, three models were terminated after fewer epochs as their learning curves did not diverge from those trained with the parameters above: Alexnet-gn-ranger-ep82, trained with the Ranger optimizer (RAdam with Lookahead, Zhang et al. (2019)); Alexnet-gn-redux-73, trained with momentum on the same cosine annealing function as the learning rate, starting at .95, dropping to .85 after 40 epochs, then rising to .95); Alexnet-gn-transforms-82, trained with transforms customized to accelerate augmentation. Although these models weren't trained for a full 100 epochs, they achieved similar top1 accuracy to models that were, and were therefore included in the primary analyses.

1.5 Assessing Emergent Categorization Accuracy

1.5.1 K-nearest neighbors evaluation

To classify a test image x, its embedding (e.g., 128 dimensional output activations) was compared to the embedding of each of the 1.28 million ImageNet training images using cosine similarity. The top $k = 200$ nearest neighbors were used to make the prediction via cosine-similarity-weighted voting, where the class c would receive the total weight given by:

$$w_c = \sum_{i}^{N_k} \exp(s_i/\tau) \cdot 1(c_i = c) \tag{6}$$

where N_k denotes the k-nearest neighbors, and s_i is the cosine similarity between the target and the neighbor, $k = 200$, and $\tau = 0.07$ (matching the value used when computing the contrastive loss during training). The target was assigned to the class with the highest weight, and scored as correct/incorrect by comparing to the ground-truth label.

1.5.2 Linear evaluation

To test whether category can be linearly decoded from model activations we trained a single fully connected layer with 1000 units on Imagenet-1k classification using the standard linear evaluation protocol (e.g., Chen et al. 2020b), in which we train a classifier on the output activations of the penultimate model layer (fc7 for Alexnet-gn models, the average pooling layer for resnet models). All parameters of the model being evaluated, including any normalization parameters, were frozen, and only the weights and biases of the fully-connected readout layer were updated. The standard linear evaluation protocol is slow and costly, training on ImageNet for 100 epochs, using stochastic gradient descent (momentum= 0.9, weight decay=0) and an initial learning rate of 30.0 which is reduced to 3.0 on epoch 60, then to .30 on epoch 80. To reduce training time we modified this standard linear evaluation protocol which enabled us to obtain similar performance levels in 10 epochs. Specifically, the learning rate was varied using the one-cycle policy (Smith, 2017), beginning at 0.00003, increasing with a cosine annealing function to a maximum of .3 after 3 epochs, then decreasing with a cosine annealing function toward zero (3e-09) by 10 epochs. We found top1 accuracy was often better, and certainly comparable with this more econimical procedure (e.g., standard vs. one-cycle top1 accuracy, 35.8% vs. 37.1% for IPCL Alexnet-gn readout from avgpool layer; 53.0% vs. 55.7% for category-supervised Alexnet-gn readout from fc7; 70.4% vs 72.0% for SWaV Resnet50 readout from the avgpool layer).

2 Supplementary Methods: fMRI

2.1 Object Orientation Dataset

2.1.1 MRI Acquisition

Imaging data were collected on a 3T Siemens Trio scanner at the Harvard University Center for Brain Sciences. Structural data were obtained in 176 axial slices with 1 x 1 x 1 mm voxel resolution, TR = 2200 ms. Functional blood oxygenation level-dependent (BOLD) data were obtained using a gradient-echo echo-planar pulse sequence (33 axial slices parallel to the anterior commissure-posterior commissure line; 70 x 70 matrix; FoV = 256 x 256 mm; 3.1 x 3.1 x 3.1 mm voxel resolution; gap thickness = 0.62 mm; TR = 2000 ms; TE = 60 ms; flip angle = 90 degrees). Volumes were acquired in ascending order. A 32-channel phased-array head coil was used.

2.1.2 Data Pre-Processing

All fMRI data was processed using Brain Voyager QX software. Preprocessing steps included 3D motion correction, slice scan-time correction, linear trend removal, temporal high-pass filtering (0.01 Hz cutoff), spatial smoothing (4mm FWHM Kernel), and transformation into Talairach space. Statistical analyses were based on the general linear model. All GLM analyses included box-car regressors for each stimulus block convolved with a gamma-function to approximate the idealized hemodynamic response. All subsequent brain-based analyses were performed using the estimated beta coefficients from the single-subject voxel-wise GLMs.

2.2 Inanimate Objects Dataset

2.2.1 MRI Acquisition

Imaging data were acquired on a BioSpin MedSpec 4T scanner (Bruker) at the University of Trentro, Italy. Functional data were collected using an echo-planar 2D imaging sequence (TR, 2000ms; TE, 33ms; flip angle, 73°; slice thickness, 3mm; gap, 0.99mm, with 3 x 3 in-plane resolution). Volumes were acquired in the axial plane parallel to the anteroposterior commissure in 34 slices, with ascending interleaved slice acquisition.

2.2.2 Data Pre-Processing

Functional data were analyzed using Brain Voyager QX software. Preprocessing included slice scan-time correction, 3D motion correction, linear trend removal, temporal high-pass filtering (0.01 Hz cutoff), spatial smoothing (6mm FWHM kernel), and transformation into Talairach (TAL) coordinates. General linear model analyses included square-wave regressors for each condition's presentation times, convolved with a gamma function to approximate the hemodynamic response. All subsequent brain-based analyses were performed using the estimated beta coefficients from the single-subject voxel-wise GLMs.

3 Supplementary Discussion

An important empirical puzzle to confront in these data is that supervision makes an extraordinary difference for accurate image classification performance, but only yields a modest difference in terms of brain fits, especially when leveraging voxel-wise encoding methods. Indeed, the success of untrained models on predicting single unit responses in IT also shows a similar pattern (e.g. Zhuang et al. (2020)). These data, and the results comparing veRSA and classic RSA indicate that encoding-model procedures for linking model and brain responses are playing a role, effectively accentuating the relevant inductive biases present in the untrained network response structure. These biases are not sufficient to enable categorization capacity without training, but do seem to initialize the representational structure in way that can be somewhat aligned with the brains representational format, particularly in early visual

cortex. Further, the group-normalization operation seems to additionally boost fits, even in classic RSA approaches.

Further post-hoc inspection of Supplementary Figure 2 reveals that the untrained models' correspondence with brain responses appears to peak in early model layers and then is sustained deeper into the network. So, interestingly, it isn't just that the max layer correspondence is high, but instead there appears to be some information that accumulates over the first few layers and is then maintained into later layers.

Given that these inductive biases seem to be fairly effective, we also explored a few untrained model variations, varying kernel sizes from .5x – 16x (relative to AlexnetGN baseline, conv1 and conv2 only), using both MaxPool and AvgPool layers. To summarize briefly, we find similar results for kernel sizes from .5x-2x, but beyond that correspondence with brain responses falls off as kernel size increases (for all brain regions in both datasets, for veRSA and classic RSA). Thus, the fits of untrained models to early visual responses appear to reflect hierarchical extraction of relatively localized structure. We have not included these analyses in the paper, because they are tangential to the primary point, but leave these breadcrumbs to satisfy the inquiring minds who find themselves reading this paragraph right now.

Supplementary Figures

```
AlexNet(
  (conv_block_1): Sequential(
    (0): Conv2d(3, 96, kernel_size=(11, 11), stride=(4, 4), padding=(2, 2), bias=False)
    (1): GroupNorm(32, 96, eps=1e-05, affine=True)
    (2): ReLU(inplace=True)
    (3): MaxPool2d(kernel_size=3, stride=2, padding=0, dilation=1, ceil_mode=False)
  )
  (conv_block_2): Sequential(
    (0): Conv2d(96, 256, kernel_size=(5, 5), stride=(1, 1), padding=(2, 2), bias=False)
    (1): GroupNorm(32, 256, eps=1e-05, affine=True)
    (2): ReLU(inplace=True)
    (3): MaxPool2d(kernel_size=3, stride=2, padding=0, dilation=1, ceil_mode=False)
  )
  (conv_block_3): Sequential(
    (0): Conv2d(256, 384, kernel_size=(3, 3), stride=(1, 1), padding=(1, 1), bias=False)
    (1): GroupNorm(32, 384, eps=1e-05, affine=True)
    (2): ReLU(inplace=True)
  )
  (conv_block_4): Sequential(
    (0): Conv2d(384, 384, kernel_size=(3, 3), stride=(1, 1), padding=(1, 1), bias=False)
    (1): GroupNorm(32, 384, eps=1e-05, affine=True)
    (2): ReLU(inplace=True)
  )
  (conv_block_5): Sequential(
    (0): Conv2d(384, 256, kernel_size=(3, 3), stride=(1, 1), padding=(1, 1), bias=False)
    (1): GroupNorm(32, 256, eps=1e-05, affine=True)
    (2): ReLU(inplace=True)
    (3): MaxPool2d(kernel_size=3, stride=2, padding=0, dilation=1, ceil_mode=False)
  )
  (avgpool): AdaptiveAvgPool2d(output_size=(6, 6))
  (fc6): Sequential(
    (0): Linear(in_features=9216, out_features=4096, bias=True)
    (1): BatchNorm1d(4096, eps=1e-05, momentum=0.1, affine=True, track_running_stats=True)
    (2): ReLU(inplace=True)
  )
  (fc7): Sequential(
    (0): Linear(in_features=4096, out_features=4096, bias=True)
    (1): BatchNorm1d(4096, eps=1e-05, momentum=0.1, affine=True, track_running_stats=True)
    (2): ReLU(inplace=True)
  )
  (fc8): Sequential(
    (0): Linear(in_features=4096, out_features=128, bias=True)
  )
  (l2norm): Normalize()
)
```

Supplementary Figure 1: *The Alexnet-gn model architecture*

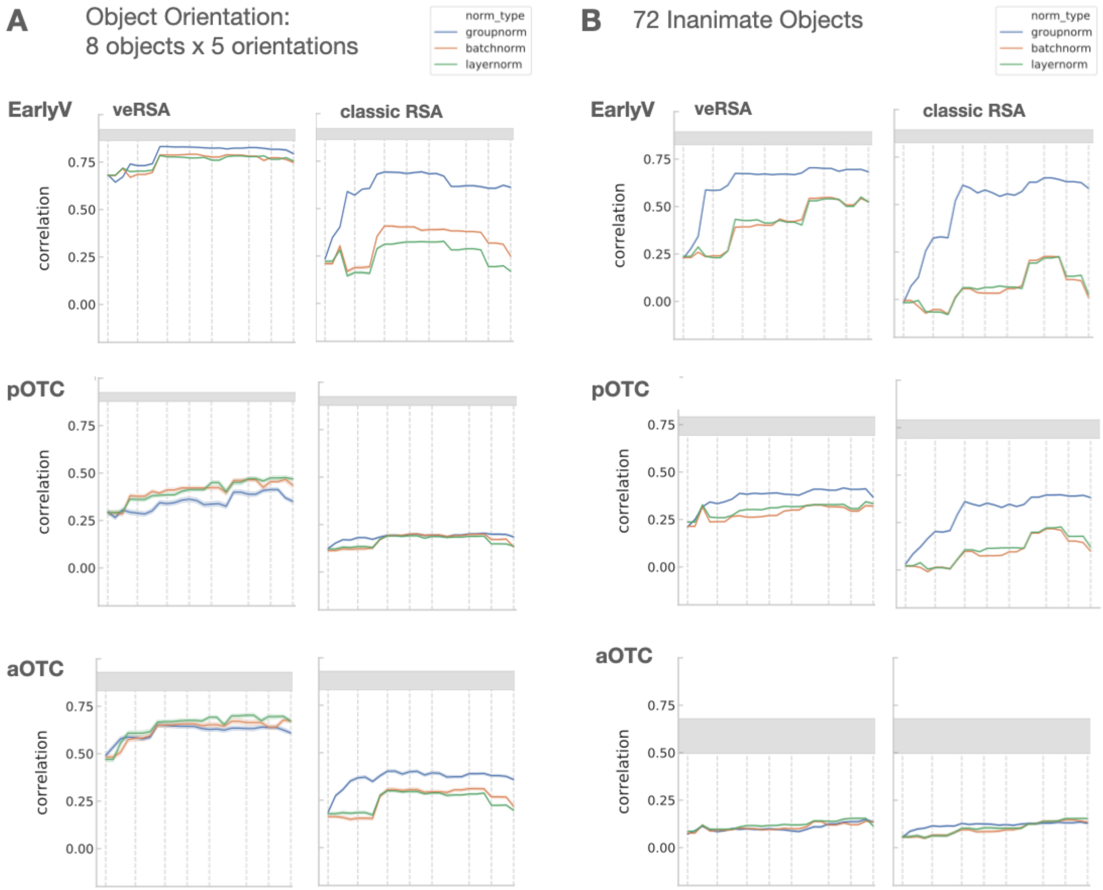

Supplementary Figure 2: *Comparison of untrained models with different normalization layers. Layerwise correlations are shown with both classic RSA and veRSA methods (y-axes), plotted as a function of model layer (x-axes), for all brain sectors (rows), and both datasets (A,B). Untrained Alexnet models with group normalization are in blue, those with batch normalization are in orange, and those with local response normalization are in green). Overall, the untrained model with group normalization layer tends to have a stronger correspondance with the brain data, compared to models with other normalization schemes. Source data are provided as a Source Data file.*

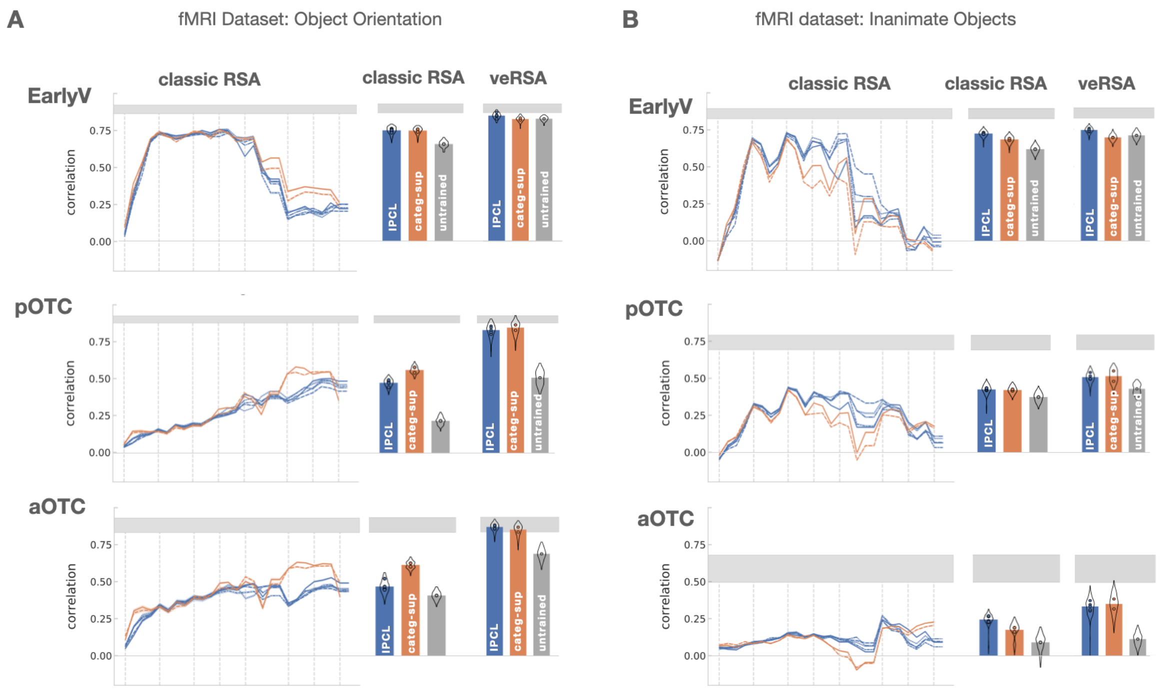

Supplementary Figure 3: *Model-Brain correspondence using Classic RSA. Layerwise correlations (y-axis) are plotted as a function of model layer (x-axis), for each brain sector (rows), in both datasets (A, B). IPCL models are in blue; Category-Supervised models in Orange. Adjacent bar-graphs plot cross-validated max correlation (y-axis) for the primary IPCL models, category-supervised models, and an untrained model. For comparison, the model-brain correlation estimated through veRSA, reported in Figure 2, is also replotted here. Overall IPCL and category-supervised models achieve similar fits to the neural data, with the exception of later model layers in pOTC and aOTC for the Object Orientation dataset. Error bars reflect a mirrored density plot (violin plot) showing the distribution of correlations across all split-halves, aggregated across instances of a given model type. Distributions are cutoff at* ± 1.5 *IQR (interquartile range, Q3-Q1). Source data are provided as a Source Data file.*

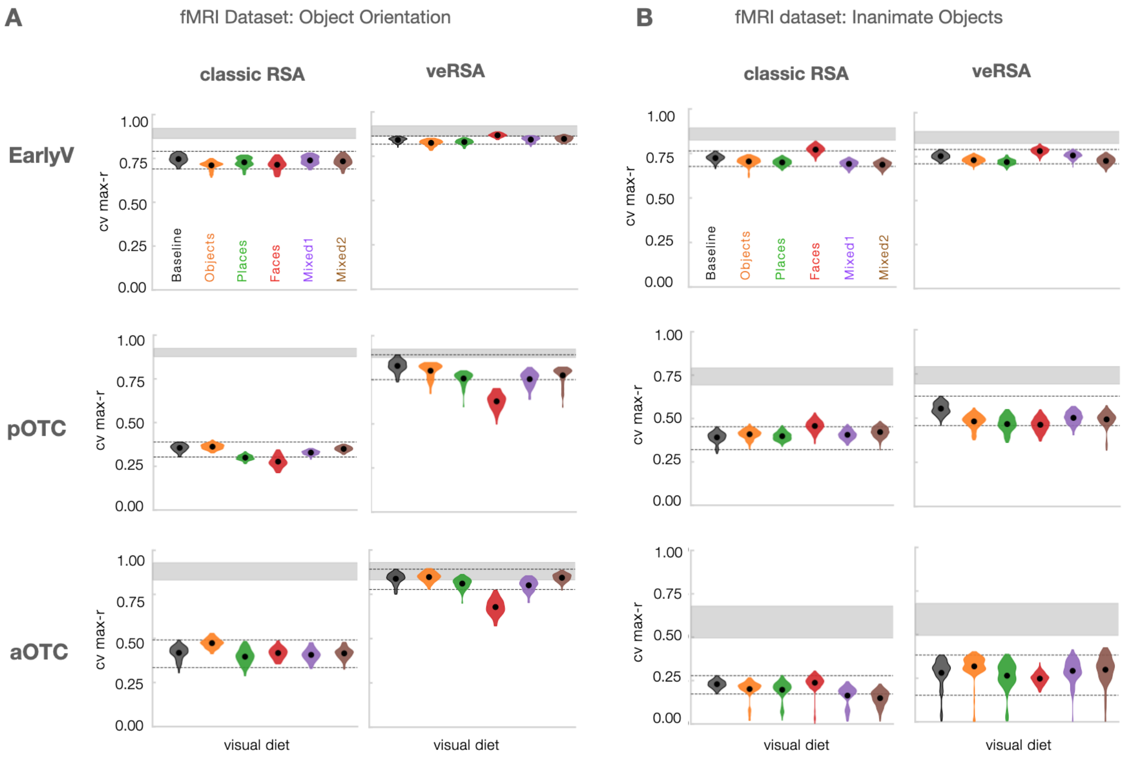

Supplementary Figure 4: *Visual Diet Variations, comparing classic RSA and veRSA. Cross-validated max-r (y-axes) was computed both with classic RSA and veRSA, in all three brain sectors (rows), and both datasets (A,B). IPCL models were trained on imagesets consisting of objects, places, faces, or mixed sets (indicated with color). Mean scores are shown with a black dot at the center of a mirrored density plot (violin plot) showing the distribution of correlations across all split-halves (distributions are cutoff at* ± 1.5 *IQR, interquartile range, Q3-Q1). The dashed black lines indicate the* ± 1.5 *IQR range for the matched baseline IPCL model trained on ImageNet. The correspondence between model RDMs and neural RDMs is greater for veRSA, but this benefit is muted for the face-trained models, specifically in the Object Orientation dataset. Source data are provided as a Source Data file.*

Type	Experiment	Ref#	Model Details	Aug	Top1-knn	Top1-linear
IPCL	Primary Models	1	primary model	Set 1	38.4	39.5
		2	variation: new code base	Set 1	38.4	39.7
		3	variation: one cycle lr & momentum (73 epochs)	Set 1	35.4	35.7
		4	variation: explore ranger (82 epochs)	Set 1	37.5	32.2
		5	variation: custom transforms (82 epochs)	Set 1	36.9	38.5
	Visual Diet	6	ImageNet baseline with new augmentations	Set 2	35.1	—
		7	train on independent object dataset, OpenImagesV6	Set 2	33.3	—
		8	train on scene dataset, Places2	Set 2	30.9	—
		9	train on face dataset, VggFace2	Set 2	12.4	—
		10	train on faces-places-objects-1x-ImageNet	Set 2	31.6	—
		11	train on faces-places-objects-3x-ImageNet	Set 2	33.9	—
Category Supervised	Primary Models	12	trained with 5 augmentations per image to match IPCL	Set 1	58.8	55.7
		13	trained with single augmentation per image	Set 1	55.5	54.5
	Visual Diet	14	ImageNet baseline with new augmentations	Set 2	56.0	—
		15	train on independent object dataset, OpenImagesV6	Set 2	56.0	—
Untrained	—	16	untrained model with random weights and biases	—	3.5 $\pm$.2	7.2

***Supplementary Table 1: Categorization Accuracy in All Trained Models**. Top1-knn classification accuracy (percent correct) is based on readout from the final layer of IPCL and untrained models (fc8), and the penultimate layer (fc7) of category-supervised models (which show higher linear readout from the penultimate layer than the final layer, as is often observed with these protocols; Chen et al., 2020a). For the untrained model, Top1-kNN shows the mean and standard deviation across 10 untrained models. Top1-linear evaluation accuracy is based on linear readout from the penultimate layer (fc7) for all models. Source data are provided as a Source Data file.*

Brain Dataset	Brain Region	Model	vs. categ-sup-12	vs. categ-sup-13	vs. untrained
Object Orientation	EarlyV	IPCL #1	$t(69)$=1.70, p=0.093	$t(69)$=0.85, p=0.400	$t(69)$=1.03, p=0.305
		IPCL #2	$t(69)$=1.63, p=0.107	$t(69)$=0.89, p=0.378	$t(69)$=1.07, p=0.288
		IPCL #3	$t(69)$=0.52, p=0.602	$t(69)$=-0.40, p=0.689	$t(69)$=-0.09, p=0.930
		IPCL #4	**$t(69)$=3.43, p=0.001**	$t(69)$=2.69, p=0.009	$t(69)$=2.99, p=0.004
		IPCL #5	$t(69)$=1.90, p=0.061	$t(69)$=1.11, p=0.271	$t(69)$=1.36, p=0.177
	pOTC	IPCL #1	$t(69)$=-2.17, p=0.033	$t(69)$=-0.87, p=0.385	**$t(69)$=3.48, p=0.001**
		IPCL #2	$t(69)$=-0.34, p=0.732	$t(69)$=1.35, p=0.180	**$t(69)$=4.80, $p<0.001$**
		IPCL #3	$t(69)$=-1.24, p=0.219	$t(69)$=0.05, p=0.957	**$t(69)$=5.12, $p<0.001$**
		IPCL #4	$t(69)$=-0.56, p=0.574	$t(69)$=0.51, p=0.614	**$t(69)$=6.62, $p<0.001$**
		IPCL #5	$t(69)$=-1.89, p=0.063	$t(69)$=-0.48, p=0.630	**$t(69)$=5.50, $p<0.001$**
	aOTC	IPCL #1	$t(69)$=1.39, p=0.169	$t(69)$=0.34, p=0.733	**$t(69)$=4.71, $p<0.001$**
		IPCL #2	$t(69)$=0.58, p=0.561	$t(69)$=-0.65, p=0.517	**$t(69)$=4.41, $p<0.001$**
		IPCL #3	$t(69)$=0.77, p=0.446	$t(69)$=-0.56, p=0.577	**$t(69)$=4.33, $p<0.001$**
		IPCL #4	$t(69)$=1.95, p=0.055	$t(69)$=1.14, p=0.256	**$t(69)$=6.13, $p<0.001$**
		IPCL #5	$t(69)$=1.86, p=0.068	$t(69)$=0.97, p=0.334	**$t(69)$=5.92, $p<0.001$**
Inanimate Objects	EarlyV	IPCL #1	**$t(251)$=3.52, p=0.001**	**$t(251)$=3.88, $p<0.001$**	**$t(251)$=3.66, $p<0.001$**
		IPCL #2	$t(251)$=2.02, p=0.044	$t(251)$=2.08, p=0.038	$t(251)$=1.96, p=0.051
		IPCL #3	$t(251)$=2.24, p=0.026	$t(251)$=2.45, p=0.015	$t(251)$=1.89, p=0.060
		IPCL #4	$t(251)$=2.58, p=0.010	$t(251)$=2.79, p=0.006	$t(251)$=2.46, p=0.015
		IPCL #5	$t(251)$=2.79, p=0.006	**$t(251)$=3.20, $p<0.0016$**	$t(251)$=2.15, p=0.032
	pOTC	IPCL #1	$t(251)$=0.59, p=0.556	$t(251)$=-2.37, p=0.019	$t(251)$=2.17, p=0.031
		IPCL #2	$t(251)$=0.45, p=0.655	$t(251)$=-2.11, p=0.035	$t(251)$=1.92, p=0.056
		IPCL #3	$t(251)$=1.11, p=0.266	$t(251)$=-1.27, p=0.204	$t(251)$=2.47, p=0.014
		IPCL #4	$t(251)$=2.91, p=0.004	$t(251)$=-0.60, p=0.546	**$t(251)$=3.93, $p<0.001$**
		IPCL #5	$t(251)$=0.70, p=0.483	$t(251)$=-2.65, p=0.009	$t(251)$=2.45, p=0.015
	aOTC	IPCL #1	$t(251)$=-0.22, p=0.826	$t(251)$=-1.12, p=0.262	$t(251)$=2.28, p=0.024
		IPCL #2	$t(251)$=0.53, p=0.594	$t(251)$=-0.91, p=0.361	**$t(251)$=3.47, p=0.001**
		IPCL #3	$t(251)$=-0.11, p=0.915	$t(251)$=-1.43, p=0.153	**$t(251)$=3.30, p=0.001**
		IPCL #4	$t(251)$=1.15, p=0.252	$t(251)$=-0.30, p=0.763	**$t(251)$=3.59, $p<0.001$**
		IPCL #5	$t(251)$=0.33, p=0.741	$t(251)$=-0.90, p=0.368	**$t(251)$=3.22, p=0.001**

Supplementary Table 2: Statistics of Primary IPCL Models. *Comparison of IPCL against the category-supervised and untrained models presented in Figure 2. Each of the five IPCL models (#1-5 in Table S1) were compared to the category-supervised models (#12,13 in Table S1), and an untrained model. Paired two-sided t-tests were computed on cross-validated max correlation values across all possible split-halves of the data (df = number of splits - 1), with a correction for non-independence of the samples (see Methods). Comparisons that were significant at the bonferroni corrected α level of .05/30=0.0017 are shown in bold. Positive t-values indicate greater predictivity for the IPCL model. Source data are provided as a Source Data file.*

Brain Dataset	Brain Region	Training Dataset	cv-max-r	fisher-z	delta-r	delta-z	z-score	adjusted t-test
Object Orientation	EarlyV	*ImageNet (baseline)*	0.84	1.23	–	–	–	–
		OpenImagesV6	0.83	1.18	−0.02	−0.05	0.15	t(69)=−1.65, p=0.104
		Places2	0.83	1.19	−0.01	−0.04	0.28	t(69)=−1.29, p=0.200
		VGGFace2	0.87	1.33	0.03	0.10	1.40	t(69)=2.46, p=0.016
		FacesPlacesObjects-1x	0.84	1.24	0.00	0.01	0.66	t(69)=0.23, p=0.817
		FacesPlacesObjects-3x	0.85	1.25	0.01	0.02	0.77	t(69)=0.54, p=0.588
	pOTC	*ImageNet (baseline)*	0.83	1.19	–	–	–	–
		OpenImagesV6	0.80	1.11	−0.03	−0.08	−0.10	t(69)=−1.09, p=0.279
		Places2	0.76	0.99	−0.07	−0.20	−1.06	t(69)=−2.21, p=0.030
		VGGFace2	0.63	0.74	−0.20	−0.45	**−3.16**	**t(69)=−5.32, p<0.001**
		FacesPlacesObjects-1x	0.76	0.99	−0.08	−0.21	−1.11	t(69)=−2.84, p=0.006
		FacesPlacesObjects-3x	0.78	1.04	−0.05	−0.15	−0.66	t(69)=−1.58, p=0.119
	aOTC	*ImageNet (baseline)*	0.84	1.22	–	–	–	–
		OpenImagesV6	0.85	1.25	0.01	0.03	0.87	t(69)=0.31, p=0.757
		Places2	0.81	1.13	−0.03	−0.09	−0.13	t(69)=−0.84, p=0.402
		VGGFace2	0.68	0.83	−0.16	−0.39	**−2.64**	**t(69)=−4.27, p<0.001**
		FacesPlacesObjects-1x	0.80	1.11	−0.04	−0.11	−0.34	t(69)=−1.10, p=0.276
		FacesPlacesObjects-3x	0.84	1.24	0.00	0.02	0.74	t(69)=0.17, p=0.869
Inanimate Objects	EarlyV	*ImageNet (baseline)*	0.75	0.98	–	–	–	–
		OpenImagesV6	0.73	0.93	−0.02	−0.05	0.18	t(251)=−1.64, p=0.103
		Places2	0.72	0.91	−0.03	−0.07	−0.02	t(251)=−2.65, p=0.009
		VGGFace2	0.78	1.05	0.03	0.07	1.18	t(251)=1.59, p=0.112
		FacesPlacesObjects-1x	0.76	0.99	0.00	0.01	0.67	t(251)=0.25, p=0.802
		FacesPlacesObjects-3x	0.73	0.92	−0.03	−0.06	0.11	t(251)=−1.54, p=0.126
	pOTC	*ImageNet (baseline)*	0.55	0.62	–	–	–	–
		OpenImagesV6	0.48	0.53	−0.07	−0.10	−0.22	t(251)=−2.14, p=0.033
		Places2	0.47	0.51	−0.09	−0.12	−0.38	t(251)=−2.92, p=0.004
		VGGFace2	0.46	0.50	−0.09	−0.12	−0.43	**t(251) = −3.40, p=0.001**
		FacesPlacesObjects-1x	0.50	0.55	−0.05	−0.07	0.00	t(251)=−2.21, p=0.028
		FacesPlacesObjects-3x	0.49	0.54	−0.06	−0.08	−0.10	t(251)=−1.86, p=0.064
	aOTC	*ImageNet (baseline)*	0.28	0.28	–	–	–	–
		OpenImagesV6	0.32	0.33	0.04	0.04	0.96	t(251)=0.58, p=0.559
		Places2	0.26	0.27	−0.01	−0.01	0.49	t(251)=−0.19, p=0.851
		VGGFace2	0.25	0.25	−0.03	−0.03	0.35	t(251)=−0.28, p=0.780
		FacesPlacesObjects-1x	0.29	0.30	0.01	0.02	0.72	t(251)=0.16, p=0.872
		FacesPlacesObjects-3x	0.30	0.31	0.02	0.03	0.81	t(251)=0.25, p=0.801

Supplementary Table 3: Statistics of Visual Diet Manipulation. *Comparison of models trained with different visual diets against the ImageNet baseline model, presented in Figure 3. The cross-validated maximum correlation (cv-max-r) scores were fisher-z transformed for statistical analyses. The difference from baseline was computed both for cv-max-r (delta-r), as well as the fisher-z transformed values (delta-z). To quantify the magnitude of differences from the baseline, the mean and standard deviation of these delta-z values was computed and a z-score was calculated (z-score). The VGGFace2 models were outliers in their difference from baseline (z-scores >2.5 SD from the mean). We also performed paired two-sided t-tests across all split-halves of the data, comparing each model and the corresponding ImageNet baseline, and adjusting for the non-independence of the samples (see Methods). Only VGGFace2-trained networks were statistically significant different from the ImageNet baseline at the Bonferroni corrected α level of .05/30=0.0017. Source data are provided as a Source Data file.*

References

Chen, T., Kornblith, S., Norouzi, M., and Hinton, G. (2020a). A simple framework for contrastive learning of visual representations. *arXiv preprint arXiv:2002.05709*.

Chen, X., Fan, H., Girshick, R., and He, K. (2020b). Improved baselines with momentum contrastive learning. *arXiv preprint arXiv:2003.04297*.

Gutmann, M. and Hyvärinen, A. (2010). Noise-contrastive estimation: A new estimation principle for unnormalized statistical models. In *Proceedings of the Thirteenth International Conference on Artificial Intelligence and Statistics*, pages 297–304.

Krizhevsky, A. (2014). One weird trick for parallelizing convolutional neural networks. *arXiv preprint arXiv:1404.5997*.

Krizhevsky, A., Sutskever, I., and Hinton, G. E. (2012). Imagenet classification with deep convolutional neural networks. In *Advances in neural information processing systems*, pages 1097–1105.

Smith, L. N. (2017). Cyclical learning rates for training neural networks. In *2017 IEEE Winter Conference on Applications of Computer Vision (WACV)*, pages 464–472. IEEE.

Wu, Y. and He, K. (2018). Group normalization. In *Proceedings of the European Conference on Computer Vision (ECCV)*, pages 3–19.

Wu, Z., Xiong, Y., Yu, S. X., and Lin, D. (2018). Unsupervised feature learning via non-parametric instance discrimination. In *Proceedings of the IEEE Conference on Computer Vision and Pattern Recognition*, pages 3733–3742.

Zhang, M. R., Lucas, J., Hinton, G., and Ba, J. (2019). Lookahead optimizer: k steps forward, 1 step back. *arXiv preprint arXiv:1907.08610*.

Zhuang, C., She, T., Andonian, A., Mark, M. S., and Yamins, D. (2020). Unsupervised learning from video with deep neural embeddings. In *Proceedings of the IEEE/CVF Conference on Computer Vision and Pattern Recognition*, pages 9563–9572.
