# Supplementary material for: A self-supervised domain-general learning framework for human ventral stream representation
Source: Nat Commun. 2022 Jan 25;13:491. doi: 10.1038/s41467-022-28091-4 (PMC8789817; doi:10.1038/s41467-022-28091-4)
Supplement: Supplementary file 3 — Reporting Summary [file 41467_2022_28091_MOESM3_ESM.pdf]

nature portfolio

Corresponding author(s): *Double-blind peer review submissions: write DBPR and your manuscript number here instead of author names.*

Last updated by author(s): *YYYY-MM-DD*

Reporting Summary

Nature Portfolio wishes to improve the reproducibility of the work that we publish. This form provides structure for consistency and transparency in reporting. For further information on Nature Portfolio policies, see our Editorial Policies and the Editorial Policy Checklist.

Statistics

For all statistical analyses, confirm that the following items are present in the figure legend, table legend, main text, or Methods section.

n/a	Confirmed	
☐	☒	The exact sample size (*n*) for each experimental group/condition, given as a discrete number and unit of measurement
☐	☒	A statement on whether measurements were taken from distinct samples or whether the same sample was measured repeatedly
☐	☒	The statistical test(s) used AND whether they are one- or two-sided<br>*Only common tests should be described solely by name; describe more complex techniques in the Methods section.*
☒	☐	A description of all covariates tested
☐	☒	A description of any assumptions or corrections, such as tests of normality and adjustment for multiple comparisons
☐	☒	A full description of the statistical parameters including central tendency (e.g. means) or other basic estimates (e.g. regression coefficient) AND variation (e.g. standard deviation) or associated estimates of uncertainty (e.g. confidence intervals)
☐	☒	For null hypothesis testing, the test statistic (e.g. *F*, *t*, *r*) with confidence intervals, effect sizes, degrees of freedom and *P* value noted<br>*Give P values as exact values whenever suitable.*
☒	☐	For Bayesian analysis, information on the choice of priors and Markov chain Monte Carlo settings
☒	☐	For hierarchical and complex designs, identification of the appropriate level for tests and full reporting of outcomes
☐	☒	Estimates of effect sizes (e.g. Cohen's *d*, Pearson's *r*), indicating how they were calculated

Our web collection on statistics for biologists contains articles on many of the points above.

Software and code

Policy information about availability of computer code

Data collection	fMRI protocols were presented using scripts written in Matlab (version 2012) using the PsychToolbox (version 2.0). For Experiment 1, the order of conditions was determined using OptSeq2 (https://surfer.nmr.mgh.harvard.edu/optseq/).
Data analysis	All analyses were conducted Python (version 3.6) using custom analysis code (https://github.com/harvard-visionlab/open_ipcl)

For manuscripts utilizing custom algorithms or software that are central to the research but not yet described in published literature, software must be made available to editors and reviewers. We strongly encourage code deposition in a community repository (e.g. GitHub). See the Nature Portfolio guidelines for submitting code & software for further information.

Data

Policy information about availability of data

All manuscripts must include a data availability statement. This statement should provide the following information, where applicable:

- Accession codes, unique identifiers, or web links for publicly available datasets
- A description of any restrictions on data availability
- For clinical datasets or third party data, please ensure that the statement adheres to our policy

Brain data, analysis code, and figure-plotting code are available on the Open Science Framework (https://osf.io/trne8/). Trained models and code are available at https://github.com/harvard-visionlab/open_ipcl.

Public image datasets used to train the models include ImageNet (https://image-net.org/), OpenImagesV6 (https://storage.googleapis.com/openimages/web/index.html), VggFace2 (https://github.com/ox-vgg/vgg_face2), and Places2 (http://places2.csail.mit.edu/).

March 2021

Field-specific reporting

Please select the one below that is the best fit for your research. If you are not sure, read the appropriate sections before making your selection.

☐ Life sciences ☒ Behavioural & social sciences ☐ Ecological, evolutionary & environmental sciences

For a reference copy of the document with all sections, see nature.com/documents/nr-reporting-summary-flat.pdf

Behavioural & social sciences study design

All studies must disclose on these points even when the disclosure is negative.

Study description	Quantitative FMRI brain data were collected
Research sample	The first fMRI experiment includes data from 7 participants (ages 20-35, 4 female, unknown racial distribution), who were recruited from the Department of Psychology study pool at Harvard University and provided written informed consent. The second fMRI dataset includes data from 10 participants (ages 19-32; 8 females, unknown racial distribution), who were recruited from the student community at the University of Trento, Italy and provided written informed consent. These study samples reflect a sample of convenience from the local communities, with the rationale that we are measuring basic, and relatively stable, visual system responses.
Sampling strategy	The first fMRI experiment includes data from 7 participants (ages 20-35, 4 female, unknown racial distribution), who were recruited from the Department of Psychology study pool at Harvard University and provided written informed consent. The second fMRI dataset includes data from 10 participants (ages 19-32; 8 females, unknown racial distribution), who were recruited from the student community at the University of Trento, Italy and provided written informed consent. These study samples reflect a sample of convenience from the local communities, with the rationale that we are measuring basic, and relatively stable, visual system responses.<br><br>Pilot studies were run before hand varying the condition rich design, to find a design that yielded reliable data in single subjects. Following this the sample size was set at 8 for Experiment 1 and 10 for Experiment 2 following conventions of the time (datasets collected in 2013 respectively). No further considerations were taken into account when selecting the sample size.
Data collection	Experiment 1: Imaging data were collected on a 3T Siemens Trio scanner at the Harvard University Center for Brain Sciences; Experiment 2: Imaging data were acquired on a BioSpin MedSpec 4T scanner (Bruker) at the University of Trentro, Italy. No one was present in the scanning room at the time of scanning besides the participant.
Timing	Data collection happened in 7/4/2013-12/2/2013 (Experiment 1) and 7/6/2013-9/13/2013 (Experiment 2)
Data exclusions	5 participants were excluded because the reliability of the visual responses (across conditions in odd/even runs) was excessively low, and/or because of excessive head motion (2 participants were from Experiment 1 and 3 from Experiment 2).
Non-participation	No participants declined to participate or otherwise dropped out of the study.
Randomization	In the fMRI scanning protocols, the order of image blocks presented in the fMRI protocols were randomized in each run, so that the exact order of image presentations was variable across participants.

Reporting for specific materials, systems and methods

We require information from authors about some types of materials, experimental systems and methods used in many studies. Here, indicate whether each material, system or method listed is relevant to your study. If you are not sure if a list item applies to your research, read the appropriate section before selecting a response.

Materials & experimental systems

n/a	Involved in the study	
☒	☐	Antibodies
☒	☐	Eukaryotic cell lines
☒	☐	Palaeontology and archaeology
☒	☐	Animals and other organisms
☐	☒	Human research participants
☒	☐	Clinical data
☒	☐	Dual use research of concern

Methods

n/a	Involved in the study	
☒	☐	ChIP-seq
☒	☐	Flow cytometry
☐	☒	MRI-based neuroimaging

Human research participants

Policy information about studies involving human research participants

Population characteristics	See Above.
Recruitment	Participants were recruited from the Department of Psychology at Harvard University through the study pool website, or from the student community at the University of Trento, Italy through the participant email list.
Ethics oversight	Experiment 1: Internal Review Board at Harvard University. Experiment 2: Internal Review Board at University of Trento.

Note that full information on the approval of the study protocol must also be provided in the manuscript.

Magnetic resonance imaging

Experimental design

Design type	Task; mini-blocked design.
Design specifications	Expt 1: Images of 8 items were presented at 5 different in-plane orientations (0, 45, 90, 135 and 180 degrees), yielding a total of 40 image conditions. These images were presented in a mini-blocked design, where in each 6min-12s run, each image was flashed 4 times (600ms on, 400ms off) in a 4s block, and was followed by 4s fixation. All 40 conditions were presented in each run; the order was determined using the optseq2 software, and was additionally constrained so that no item appeared in consecutive blocks (e.g. an upright dog, followed by an inverted dog). Two additional 20s rest periods were distributed throughout the run. Participants completed 12 runs. Their task was to pay attention to each image and complete a vigilance task (press a button when a red circle appeared around an object), which happened 12 times in run.<br><br>Expt 2: Images of 72 inanimate items were presented. In each 8-min run, each image was flashed 4 times (600ms on, 400ms off) in a 4s block, with all 72 images presented in a block in each run (randomly ordered), with 4×15s rest periods interleaved throughout. Participants completed 6 runs. Their task was to pay attention to each image and complete a vigilance task (press a button when a red-frame appeared around an object, which happened 12 times in run).
Behavioral performance measures	Participants' task was to pay attention to each image and press a button when an low frequency red circle appeared around an object. Keypresses were monitored during scanning, to help ensure participants had not fallen alseep. Performance was not subsequently analyzed.

Acquisition

Imaging type(s)	functional
Field strength	3T (expt 1), 4T (Expt 2)
Sequence & imaging parameters	Expt 1: unctional blood oxygenation level-dependent (BOLD) data were obtained using a gradient-echo echo-planar pulse sequence (33 axial slices parallel to the anterior commissure-posterior commissure line; 70 x 70 matrix; FoV = 256 x 256 mm; 3.1 x 3.1 x 3.1 mm voxel resolution; gap thickness = 0.62 mm; TR = 2000 ms; TE = 60 ms; flip angle = 90 degrees).<br><br>Expt 2: Functional data were collected using an echo-planar 2D imaging sequence (TR, 2000ms; TE, 33ms; flip angle, 73°; slice thickness, 3mm; gap, 0.99mm, with 3 x 3 in-plane resolution).
Area of acquisition	whole-brain

Diffusion MRI ☐ Used ☒ Not used

Preprocessing

Preprocessing software	Brain Voyager QX software (version unknown)
Normalization	Normalization
Normalization template	TAL
Noise and artifact removal	none.
Volume censoring	none.

Statistical modeling & inference

Model type and settings	mass univariate

Effect(s) tested

Betas were extracted from the single subject GLMs and were used in second level analyses relating deep neural network model features to voxel-wise activations and regional multivoxel pattern similarity structure.

Specify type of analysis: ☐ Whole brain ☒ ROI-based ☐ Both

Anatomical location(s)

First, the EarlyV sector was defined for each individual to include areas V1-V3, which were delineated based on activations from a separate retinotopy protocol. Next, an occipitotem- poral cortex mask was drawn by hand on each hemisphere (excluding the EarlyV sector), within which the 1000-most active voxels were included, based on the contrast [all objects > rest] at the group-level. To divide this cortex into posterior and anterior OTC sectors, we used an anatomical cut off (TAL Y:
-53), based on a systematic dip in local-regional reliability at a this anatomical location, based off of concurrent work also analyzing this Inanimate Object dataset (Magri and Konkle, 2020). The same posterior-anterior division was applied to define the sectors and extract data from the Object Orientation dataset

Statistic type for inference
(See Eklund et al. 2016)

To compare the cross-validated max correlation values between models, we used paired t-tests over all split halves of the data, with a correction for non-independence of the samples, following Bouckaert and Frank, 2004 (tests based on repeated k-fold cross validation) for corrected variance estimate and adjusted t-values.

Correction

Statistical significance for these paired t-tests was determined using a Bonferonni corrected α level of .05/30=0.00167, where 30 corresponds to the number of family-wise tests for all reported tests.

Models & analysis

n/a	Involved in the study
☒	☐ Functional and/or effective connectivity
☒	☐ Graph analysis
☐	☒ Multivariate modeling or predictive analysis

Multivariate modeling and predictive analysis

Voxelwise Encoding. For each deepnet layer, subject, and sector, each voxel's response profile (over 40 or 72 image conditions, depending on the dataset) was fit with an encoding model. Specifically, in a leave-one-out procedure, a single image was held out, and ridge regression was used to find the optimal weights for predicting each voxel responses to the remaining images. We used sklearn's (Pedregosa et al., 2011) cross-validated ridge regression to find the optimal lambda parameter. The response for the held-out item was then predicted using the learned regression weights. Each item was held out once, providing a cross-validated estimate of responses to each image in every voxel, which together form a model-based prediction of neural responses in each brain region. Based on these predicted responses, a model-predicted-RDMs was computed for each participant.

Layerwise RSA analysis. Next, for each sector and layer, the model-predicted-RDMs for each subject were divided into two groups and averaged, yielding two average model-predicted-RDMs from two independent halves of the data. Each RDM was correlated with actual brain-RDM, where the brain-RDM was computed from the same set of participants. This analysis was repeated for all possible splits-halves of the participants. The average fisher-z transformed correlation (and an adjusted 95% confidence interval Bouckaert and Frank 2004) was taken as the key measure of layer-sector correspondence.

Note that this average correlation reflects the similarity between the model-predicted-RDMs and the brain-RDMs, where only half of the subject's brain data are used. This method of splitting the data into two halves was designed to increase the reliability in the data—we found that the RDMs were more stable with the benefit of averaging across subjects, while any one individual's brain data was generally less reliable. Additionally, this procedure allows there to be some generality across subjects. Finally, we did not adjust the fit values to correct for the fact that the model-to-brain fit reflects only half the brain data, instead we kept it as is, which also allows the average layer-sector correlation to be directly compared to the similarly-estimated noise ceiling of the brain data.

Cross-Validated Max-Layer Estimation. The second key dependent measure relating model-brain correspondence reflects the strength of the best-fitting layer to a given sector. To compute this measure, we again used the same technique of splitting the data in half by two groups of subjects (this time to prevent double-dipping). Specifically, for each model and sector, the veRSA correlation was computed for all layers, and the layer with the highest veRSA correlation was selected. Then, in the independent half of the data (from new participants), the veRSA correlation was computed for this selected layer, and taken as a measure of the highest correspondence between the model and the sector. As above, this procedure was repeated for all possible split-halves of the subjects, and the cross-validated max-r measure was taken as the average across splits (averaging fisher-z transformed correlation values, and using the adjusted 95% confidence interval that takes into account the non-independence of the samples). This procedure insures an independent estimate of the maximum correspondence across layers.
